# Relationship between testosterone-estradiol ratio and some anthropometric and metabolic parameters among Nigerian men

**DOI:** 10.1016/j.metop.2023.100249

**Published:** 2023-06-05

**Authors:** Holiness Stephen Adedeji Olasore, Tolulope Adejoke Oyedeji, Matthew Olamide Olawale, Omobolanle Ibukun Ogundele, Joseph Ogo-Oluwa Faleti

**Affiliations:** a Department of Biochemistry, Faculty of Basic Medical Sciences, College of Medicine of the University of Lagos, Idi Araba, Lagos, Nigeria; b Department of Pharmacognosy, Faculty of Pharmacy, University of Lagos, Idi Araba Campus, Lagos, Nigeria

**Keywords:** Testosterone, Estradiol, Weight, Height, Body mass index, Fasting blood glucose, Plasma lipid profile

## Abstract

**Background:**

Alterations in sex hormone levels are implicated in the regulation of metabolic processes in men. In recent years, the prevalence of metabolic disorders, such as obesity, insulin resistance, and type 2 diabetes, has risen in Nigeria. In men, these disorders may be associated with the ratio of serum testosterone to estradiol levels. Therefore, we investigated the relationship between the testosterone-estradiol (T/E2) ratio, anthropometry, and metabolic parameters in Nigerian men.

**Method:**

Eighty-five adult men were recruited for this study. Participants’ data such as age, weight, height, BMI, and waist circumference were collected. Plasma total testosterone and estradiol levels, as well as metabolic parameters such as fasting blood sugar, creatinine, urea, HDL cholesterol, total cholesterol, and triglycerides levels, were determined. The data were analyzed using SPSS version 25 software.

**Results:**

Anthropometric parameters such as weight, height, BMI, and waist circumference showed a negative correlation with plasma T/E2 (r = −0.265, −0.288, −0.106, −0.204; p = 0.007, 0.004, 0.167, 0.061 respectively). However, the T/E2 ratio showed a positive correlation with the metabolic parameters such as fasting blood sugar, HDL cholesterol levels, plasma creatinine, and urea (r = 0.219, 0.096, 0.992, 0.152; p = 0.022, 0.192, <0.001, 0.082 respectively), while there were negative correlations with total cholesterol and triglycerides levels (r = −0.200, −0.083; p = 0.034, 0.226 respectively).

**Conclusion:**

These findings show that there are significant correlations between the T/E2 ratio and weight, height, fasting blood sugar, creatinine, and urea, while there are no significant correlations between T/E2 ratio and BMI, waist circumference, HDL-cholesterol, and triglycerides.

## 1 Introduction

Estrogens, long thought to be exclusive to women, also play a significant role in the biological functions of men [1]. Androgens and estrogens are commonly known as reproductive hormones and have also shown general metabolic functions that are not directly involved in the reproductive process [2]. Estrogen levels have long been linked to a range of metabolic functions, controlling body fat distribution, and sexual dimorphism in both sexes, with studies demonstrating their role in regulating energy expenditure, bone turnover, and metabolite levels [2,3]. The processes of spermatogenesis, erection, gonadotropin regulation, and bone mineral density in men are some of the additional roles of estrogen [4,5].

Estrogen is synthesized from testosterone by aromatase also known as estrogen synthetase; EC 1.14.14 [6]. This enzyme catalyzes the conversion of testosterone to estradiol (E2) by an oxygen-dependent and NADPH-dependent reaction [7]. Aromatase is expressed in both male and female gonads, as well as in extra-gonadal sites, such as adipose tissues, bone, placenta, and brain [8]. The measurement of testosterone to estradiol ratio in the blood has been instrumental to evaluate intracellular aromatase activity in both males and females [9], with higher ratios of testosterone to estradiol indicating lower aromatase activity. A mutation in the CYP19A gene which encodes aromatase, results in undetectable serum estradiol levels with normal or high testosterone levels [10]. There are only a few cases of aromatase deficiency in humans, but this indirect link of the measure of aromatase activity has also been strongly established in aromatase gene-knockout mouse models [11].

The activity of aromatase, resulting in an imbalance in the distribution of these sex hormones (testosterone and estradiol), is crucial in a variety of physiological processes [12]. Anthropometric parameters, like body mass index (BMI), neck circumference (NC), waist circumference, and waist-to-hip ratio (WHR), among others, have been linked with changes in sex hormone levels in both men and women across different age groups [13,14]. These measures of adiposity have been linked to increased levels of estrogen and decreased levels of testosterone in both men and women [15]. Studies have also shown that men with higher BMI are predisposed to increased estradiol levels and decreased testosterone levels [16], possibly due to increased adipocyte expression of aromatase. Furthermore, the expression of aromatase in subcutaneous adipose tissue was found to be significantly higher in individuals who are obese compared to those who are not obese [17].

Studies have shown that as total fat mass increases, resistance to metabolic hormone regulators, such as leptin and insulin, develops [18], resulting in alterations in lipid metabolism. A high estradiol-to-testosterone ratio in the blood is associated with the elevation of triglycerides, LDL cholesterol, and total cholesterol levels but a decrease in HDL cholesterol levels [19,20]. These changes in the lipid profile may result in the onset of atherosclerosis [21], a condition defined by the accumulation of plaques in the arteries, and a major risk factor for cardiovascular disease.

Understanding the association between the testosterone-estradiol ratio, anthropometric parameters, and metabolic parameters could provide insights into the underlying causes of metabolic dysfunctions as well as the development of potential prevention methods and therapies for related clinical conditions which includes obesity and cardiovascular diseases. The aim of this present study was to investigate the correlation between the testosterone-estradiol ratio, anthropometric parameters like weight, height, and BMI, and plasma metabolic parameters in men.

## 2 Materials and methods

### 2.1 Ethical consideration and subjects

This study was approved by the Health Research Ethics Committee (HREC) of the College of Medicine of the University of Lagos. All participants provided their informed consent by signing the consent form. A total of 85 adult men were recruited from the university community for the study. All participants were free from any metabolic and endocrine diseases. Questionnaires were administered to obtain data on the participants’ biodata and lifestyles.

### 2.2 Anthropometric measurements

Body weight and height were measured using a standard weighing balance and stadiometer while BMI was calculated. Waist circumference was measured with the use of a tape measure.

### 2.3 Sample collection

All participants were required to fast overnight by stopping food intake from 8 p.m. on the day prior to sample collection. Five milliliters of venous blood was taken from each participant by a trained phlebotomist after the overnight fast and a drop of whole blood was placed on the Accu-Chek® Active blood glucose strip and read with Accu-Chek® Active blood glucose meter (Roche Diabetes Care, Inc., USA) to determine the fasting blood sugar. The remaining blood sample was put into an EDTA sample bottle and was spun at 2500 rpm to obtain plasma for other biochemical assays.

### 2.4 Biochemical analyses

*Hormonal Assay:* Plasma concentrations of total estradiol and total testosterone were measured by electrochemiluminescence immunoassay using an Elecsys 2010 system (Roche Diagnostics, Diagnostic, USA).

*Lipid Profile:* The Hitachi 704 analyzer (Roche Diagnostics, USA) was used to analyze plasma cholesterol, triglyceride, and HDL-cholesterol using the recommended reagents (Roche Diagnostics, USA). A cholesterol high-performance reagent was used to analyze total cholesterol enzymatically, while direct HDL-cholesterol reagent was used to analyze HDL-cholesterol.

### 2.5 Statistical analyses

Statistical analyses were carried out using SPSS version 25 software. The descriptive statistics were presented as mean ± SD while the BMI categories were presented as frequencies and percentages. Pearson's correlations were used to analyze the correlations between the T/E2 ratio and anthropometric as well as metabolic parameters. The level of statistical significance was set at p < 0.05.

## 3 Results

Table 1 shows the description of the study participants used in this study. The participant's age, weight, height, BMI, and waist circumference presented as mean ± SD were 38.04 ± 12.24 years, 67.14 ± 9.55 kg, 1.70 ± 0.07 m, 23.34 ± 3.14 kg/m^2^, and 73.45 ± 9.13 cm respectively. Men with weights within the normal range predominated the study population with a frequency of 56 (65.9%) of the whole population sample, followed by overweight, underweight, and obese individuals making up 22 (25.1%), 5 (5.9%), and 2 (2.4%), respectively.

**Table 1:** Descriptive statistics of the population studied.

Age (mean ± SD) years	38.04 ± 12.24
Weight (mean ± SD) kg	67.14 ± 9.55
Height (mean ± SD) m	1.70 ± 0.07
BMI (mean ± SD) kg/m^2^	23.34 ± 3.14
BMI categories n (%)	
Underweight	5 (5.9)
Normal	56 (65.9)
Overweight	22 (25.1)
Obese	2 (2.4)
Waist circumference (mean ± SD) cm	73.45 ± 9.13

Table 2 shows the distribution of the various hormonal and metabolic parameters in the study population. The mean ± standard deviation (SD) for testosterone was 568.42 ± 190.67 ng/dL, while the mean ± SD for estradiol was 17.96 ± 7.76 pg/mL. The testosterone-estradiol ratio had a mean ± SD value of 3.67 ± 1.75. Other parameters measured include fasting blood sugar, creatinine, urea, HDL cholesterol, total cholesterol, and triglycerides. The fasting blood sugar level had a mean ± SD value of 94.44 ± 16.26 mg/dL. The mean values (±SD) of creatinine and urea were 89.70 ± 12.51 μmol/L and 3.73 ± 0.95 mmol/L respectively. In addition, the mean values (±SD) of total cholesterol, HDL, and triglycerides were 4.01 ± 0.93 mmol/L, 1.01 ± 0.60 mmol/L, and 1.01 ± 0.60 mmol/L respectively.

**Table 2:** Distribution of hormonal and metabolic parameters.

Analyte	Mean ± SD
Testosterone (ng/dL)	568.42 ± 190.67
Estradiol (pg/mL)	17.96 ± 7.76
Testosterone-estradiol ratio	3.67 ± 1.75
Fasting blood sugar (mg/dL)	94.44 ± 16.26
Creatinine (μmol/L)	89.70 ± 12.51
Urea (mmol/L)	3.73 ± 0.95
HDL (mmol/L)	1.39 ± 0.38
Total cholesterol (mmol/L)	4.01 ± 0.93
Triglycerides (mmol/L)	1.01 ± 0.60

**Fig. 1:**
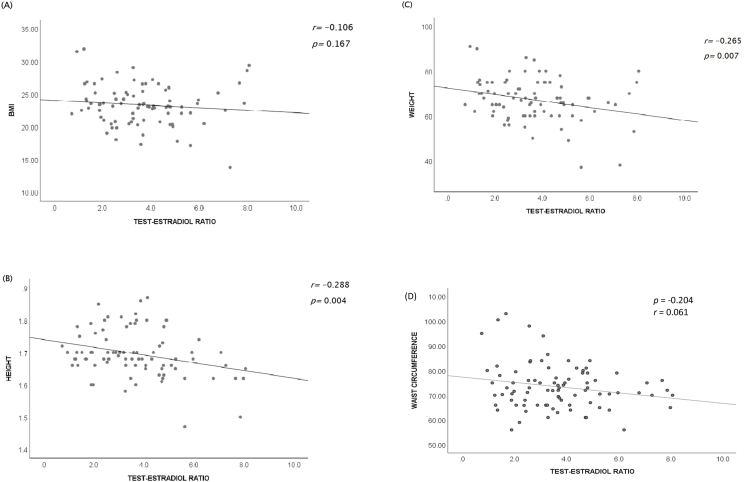
Scatterplot showing the correlation between testosterone-estradiol ratio and anthropometric parameters (A) BMI, (B) Height, (C) Weight of the study participants, and (D) Waist circumference.

The relationships between testosterone-estradiol and anthropometric parameters are presented in Fig. 1. The BMI, height, weight, and waist circumference were negatively correlated with the T/E2 ratio (r = −0.106, p = 0.167; r = −0.288, p = 0.004; r = −0.265, p = 0.007 and r = - 0.204, p = 0.061 respectively).

**Fig. 2:**
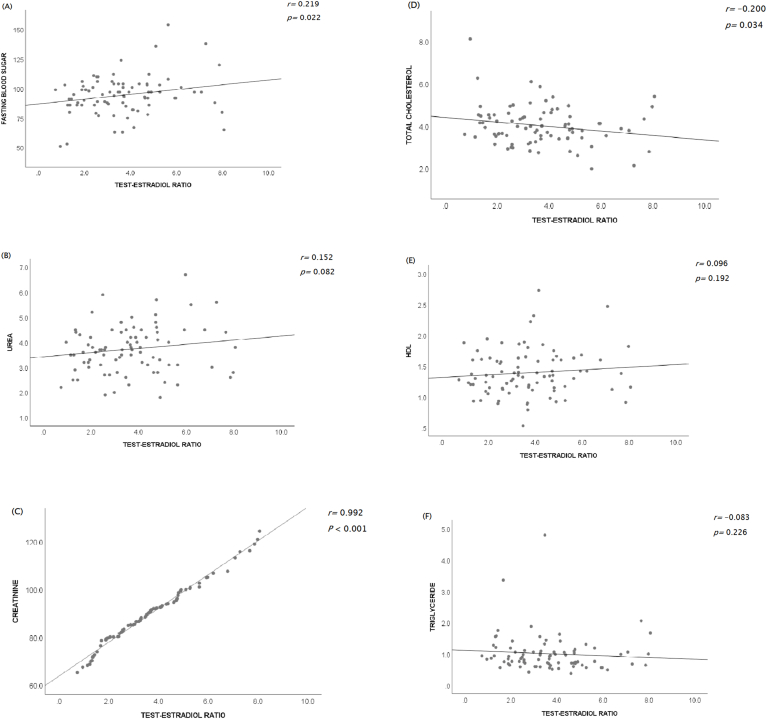
Scatterplot showing the correlation between testosterone-estradiol ratio and metabolic parameters (A) Fasting Blood Sugar, (B) Urea, (C) Creatinine, (D) Cholesterol, (E) HDL, and (F) Triglyceride of the study participants.

Fig. 2 shows a scatter plot of the relationship between the testosterone-estradiol ratio and metabolic parameters. We found a moderate positive correlation between the T/E2 ratio and fasting blood sugar level, which was statistically significant (r = 0.219, p = 0.022). The levels of serum urea and creatinine show a positive correlation with the T/E2 ratio (r = 0.152, p = 0.082 and r = 0.992, p < 0.001 respectively). In addition, the results of the lipid profiles show that the levels of HDL are positively correlated with the T/E2 ratio (r = 0.096, p = 0.192). Meanwhile, total cholesterol and triglycerides show some negative correlation with the T/E2 ratio (r = −0.200, p < 0.034 and r = −0.083, p = 0.226 respectively).

## 4 Discussion

We have examined the correlations between the testosterone-estradiol (T/E2) ratio with anthropometric parameters and the distribution of metabolic parameters in men. The present study's findings showed a negative correlation between the T/E2 ratio and anthropometric parameters such as BMI, height, weight, and waist circumference. This suggests that men with a lower T/E2 ratio may be taller with higher body weight. These findings are comparable to previous studies [22,23]. Higher estradiol levels have been associated with increased adiposity and weight in men [15]. These high estradiol levels can be accounted for by the higher conversion rates of testosterone to estradiol in adipose tissues by aromatization [24], thus, resulting in a decrease in the T/E2 ratio. Estradiol, among several hormones, has been linked to helping in the maintenance and control of lipid storage in the adipose tissues and plays a role in the prevention of the onset of obesity [25,26]. Therefore, it is logical to suggest that as the body fat increases there is a proportional increase in the estradiol levels by the action of aromatase and a relative decline in testosterone levels. However, the weak correlation found in our study between the T/E2 ratio and BMI may be explained by individual variability in the sample population.

In this present study, we found a negative correlation between the T/E2 ratio and height. In males, puberty causes an acceleration of growth due to hormonal changes and environmental factors, leading to the pubertal growth spurt. However, towards the end of puberty, growth in height may slow down or cease, as the epiphyseal plates close [27]. The increased levels of testosterone during puberty have been linked to epiphyseal plates narrowing and ultimately closing [28]. It has also been believed that testosterone levels decline as men age [29], and it is possible to maintain the height attained during puberty even with low testosterone levels at an older age. A prior study by Sartorius et al. (2012) [30] which included 325 men with an average age of 60 years, observed that older men with low testosterone levels still had a taller stature compared to men with normal or high testosterone levels.

Considering the waist circumference, we also observed that there was a negative correlation between this parameter and the T/E2 ratio. Waist circumference is more points more to fat distribution than the BMI and indicates visceral adiposity. Deposition of fat in the visceral area has been linked to adverse health effects such as increased cardiometabolic risks [23]. Reduced levels of testosterone and lower T/E2 ratio have been reported to negatively correlate to body fat and its distribution. While lower testosterone levels have been associated with more visceral fat, higher estrogen is known to favor increased visceral adiposity [23,31] This is in line with our finding as shown by the waist circumference.

It has previously been demonstrated that increased aromatase activity can have an impact on metabolic dysfunctions [32]. Prior research has found that high estradiol levels and low testosterone levels, and a lower T/E2 ratio are more prevalent in men with impaired glucose metabolism and persistence of blood glucose [33,34]. These are contrary to our findings which have shown that the direction of the relation of T/E2 ratio and fasting blood sugar is positive, suggesting that higher fasting blood sugar levels may be associated with a higher T/E2 ratio in men (low estradiol and high testosterone levels). Maintaining glucose homeostasis has been linked to estradiol by promoting glucose uptake in muscle and adipose tissue [35], and suppressing glucose production in the liver via gluconeogenesis and glycogenolysis [36]. Low doses of estradiol have been shown to enhance insulin sensitivity and promote glucose uptake in both men and women [37,38]. It is logical to say that men with low levels of estradiol may be prone to elevated fasting blood glucose.

Testosterone and estradiol have shown distinct effects on human lipoprotein metabolism [39]. Testosterone has been shown to decrease lipid synthesis but increase lipolysis, thereby lowering serum triglyceride [40] and low-density lipoprotein (LDL) cholesterol levels. Lipoprotein HDL helps to carry LDL-cholesterol from the circulation back to the liver for removal which helps in protection against the development of atherosclerosis [41]. Our findings, along with those of previous studies, show that sex hormone imbalances may play a role in the modulation of circulating lipid metabolite levels in men [42]. We observed a positive correlation between serum HDL-cholesterol and total cholesterol, suggesting that a greater T/E2 ratio in men may be related to slightly higher levels of HDL-cholesterol, but a negative correlation between the T/E2 ratio and triglycerides and total cholesterol, suggesting men with higher T/E2 ratios tend to have lower serum triglycerides and total cholesterol levels. The positive relationship between the T/E2 ratio and HDL levels has important implications for cardiovascular health, as lower HDL cholesterol levels have been linked with the risk of cardiovascular diseases [43]. Therefore, maintaining a balance between testosterone and estradiol levels in men may be critical for maintaining HDL cholesterol levels and lowering the risk of cardiovascular disease.

There was a relationship between creatinine and the T/E2 ratio in our study. It is long believed that creatinine levels show an indirect measure of muscle mass [44], since an increase in muscle mass leads to the production of creatinine during muscle metabolism. Prior research has shown that testosterone levels in men correlate with creatinine levels [45], and it is suggestive that this correlation may be due to the stimulatory effect of testosterone on muscle mass. Additionally, greater muscle mass will show high metabolic activity in the muscle, increasing the rate of muscle protein turnover and resulting in higher blood urea levels [46,47]. There is evidence that suggests that testosterone aids renal functioning by increasing the renal clearance of creatinine and urea from the blood [48,49].

The limitations of this study, include not involving women to see if similar effects could be replicated in this category of participants. Furthermore, including hormonal parameters such as the sex hormone binding globulin (SHBG) and free androgen index (FAI) could have made the study more robust. However, regardless of the limitations of this study, our findings provide insights into the possible association between the T/E2 ratio and various physiological parameters in men.

## 5 Conclusion

Generally, our findings in this study have important implications for clinical practice. We found that body weight, height, and BMI show a correlation to the T/E2 ratio which could be used indirectly to measure aromatase activity. Higher T/E2 ratios can be linked to better metabolic health, including higher HDL, lower total cholesterol, and lower circulating triglycerides which are considered markers of good metabolic status in men. This highlights the importance of monitoring these metabolic parameters in men with testosterone and estrogen imbalances. Moreover, our findings also show a correlation between the T/E2 ratio and renal function markers, such as serum creatinine and urea, suggesting that changes in the T/E2 ratio may also have an impact on renal function in men. Further research is necessary to corroborate these findings and to investigate the underlying mechanisms and interactions between the T/E2 ratio and metabolism. We also recommend that future studies should include a larger number of participants.

## Funding

This research did not receive any specific grant from funding agencies in the public, commercial, or not-for-profit sectors.

## Declaration of conflict of interest

The authors have no conflict of interest to declare for this study.

## CRediT authorship contribution statement

**Holiness Stephen Adedeji Olasore:** Conceptualization, Data curation, Formal analysis, Methodology, Supervision, Writing – review & editing. **Tolulope Adejoke Oyedeji:** Conceptualization, Methodology, Writing – review & editing. **Matthew Olamide Olawale:** Data curation, Methodology, Writing – review & editing. **Omobolanle Ibukun Ogundele:** Data curation, Formal analysis, Methodology, Writing – review & editing. **Joseph Ogo-Oluwa Faleti:** Data curation, Formal analysis, Methodology.
